# Internet Addiction Among Nursing Students and Its Associated Factors

**DOI:** 10.1192/j.eurpsy.2026.10909

**Published:** 2026-07-31

**Authors:** I. Kacem, S. Gmiza, C. Sridi, A. Aloui, F. Chelly, A. Fki, A. Chouchane, M. Maoua, M. Kahloul

**Affiliations:** 1 Occupational Medicine Department, Farhat Hached University Hospital; 2 University of Sousse, Faculty of Medicine of Sousse; 3 Research Laboratory LR19SP0; 4 Occupational Medicine Department; 5 Anesthesia and Intensive Care Department, Sahloul University Hospital, Sousse, Tunisia

## Abstract

**Introduction:**

University entrance is a critical transition for nursing students, who face intense academic, emotional, and social demands that may predispose them to maladaptive behaviors like internet addiction. The growing reliance on digital platforms, especially social media, has heightened risks of compulsive internet use among university populations. However, this phenomenon remains understudied in Tunisia, particularly within healthcare education contexts. This study therefore aims to assess the prevalence of internet addiction among Tunisian nursing students and identify its key associated factors to inform targeted support strategies.

**Objectives:**

To evaluate the prevalence of social media addiction and its associated factors among nursing students in Tunisia.

**Methods:**

A cross-sectional analytical study was conducted in March 2025 among nursing students across selected institutions in Tunisia. Participants completed a self-administered questionnaire capturing socio-demographic characteristics, lifestyle behaviors, and internet use patterns, with internet addiction assessed using the validated 20-item Internet Addiction Test (IAT). Data were analyzed to determine prevalence rates and identify factors significantly associated with internet addiction.

**Results:**

The study population was predominantly female (72.3%) with a mean age of 22 years. The prevalence of social media addiction (IAT score ≥ 31) was 54.9%. Logistic regression indicated that students with internet addiction (IAT ≥ 31) were 2.5 times more likely to sleep fewer than 6 hours per night (aOR = 2.5, 95% CI: 1.4–4.3, p = 0.003)

**Conclusions:**

This study reveals a high prevalence of social media addiction among Tunisian nursing students. Intensive smartphone use, sacrificed sleep to stay connected, and early registration on social media appear as significant risk factors. These findings highlight the urgent need to integrate preventive strategies targeting digital health.

**Disclosure of Interest:**

None Declared

